# Perchlorate exposure does not induce obesity or non-alcoholic fatty liver disease in zebrafish

**DOI:** 10.1371/journal.pone.0254500

**Published:** 2021-08-04

**Authors:** Michael R. Minicozzi, Erik G. Axlid, Frank A. von Hippel, Joseph Espinoza, Aubrey Funke, Quentin P. Phillips, C. Loren Buck

**Affiliations:** 1 Department of Biological Sciences, Minnesota State University Mankato, Mankato, MN, United States of America; 2 Department of Community, Environment and Policy, The University of Arizona, Tucson, AZ, United States of America; 3 Department of Biological Sciences, Northern Arizona University, Flagstaff, AZ, United States of America; Auburn University, UNITED STATES

## Abstract

Perchlorate is a water-soluble contaminant found throughout the United States and many other countries. Perchlorate competitively inhibits iodide uptake at the sodium/iodide symporter, reducing thyroid hormone synthesis, which can lead to hypothyroidism and metabolic syndromes. Chronic perchlorate exposure induces hepatic steatosis and non-alcoholic fatty liver disease (NAFLD) in developing threespine stickleback (*Gasterosteus aculeatus*). We hypothesized that perchlorate would also induce zebrafish (*Danio rerio*) to develop phenotypes consistent with NAFLD and to accumulate lipids throughout the body. We exposed zebrafish embryos to four concentrations of perchlorate treated water (10μg/L, 10mg/L, 30mg/L, and 100mg/L) and a control (0mg/L) over the course of 133 days. Adult zebrafish were euthanized, sectioned, H&E and Oil Red-O stained, and analyzed for liver morphology and whole body lipid accumulation. In a representative section of the liver, we counted the number of lipid droplets and measured the area of each droplet and the total lipid area. For whole body analysis, we calculated the ratio of lipid area to body area within a section. We found that zebrafish exposed to perchlorate did not differ in any measured liver variables or whole body lipid area when compared to controls. In comparison to stickleback, we see a trend that control stickleback accumulate more lipids in their liver than do control zebrafish. Differences between the species indicate that obesogenic effects due to perchlorate exposure are not uniform across fish species, and likely are mediated by evolutionary differences related to geographic location. For example, high latitude fishes such as stickleback evolved to deposit lipid stores for over-winter survival, which may lead to more pronounced obesogenic effects than seen in tropical fish such as zebrafish.

## Introduction

Obesity rates in the United States, and in many other countries around the world, have risen persistently for several decades in what is known as the global obesity epidemic [1]. The trend has profound implications for obesity-related diseases as well as economic and social structures within communities [2–4]. The view within the scientific community has long been that high-calorie diets and increasingly sedentary lifestyles are the main drivers of this trend [5]. Although these factors should not be discounted, recent evidence suggests that exposure to environmental contaminants may also influence fat accumulation in animals, including humans [6, 7]. This link between obesity and chemical factors is known as the obesogen hypothesis, and the chemicals (a class of endocrine disrupting compounds [EDCs]) that promote abnormal lipid accumulation are known as obesogens [6–9].

EDCs can disrupt normal hormone levels, and potentially affect lipid accumulation, through a number of pathways. For example, many EDCs affect the regulation of sex hormones, the hypothalamic-pituitary-thyroid (HPT) axis, or other endocrine pathways [6, 10]. Changes in androgen and estrogen levels (sex hormones) influence lipid accumulation and can cause obesity [11, 12]. For example, estrogens control subcutaneous fat accumulation [13] and xenoestrogens, such as hexachlorocyclohexanes, are linked to the development of metabolic abnormalities in obese women [14]. Many of these chemicals work indirectly through epigenetic mechanisms by affecting key genes linked to obesity. Specifically, phthalate esters such as bisphenol-a and mono-(2-ethylhexyl) phthalate upregulate adipocyte or lipogenic genes which can lead to obese phenotypes [15, 16]. Additionally, EDCs can act directly on the HPT axis by altering levels of circulating thyroid hormones (triiodothyronine [T_3_] and thyroxine [T_4_]) which regulate, among other things, lipid metabolism [6]. Hypothyroidism is linked to reduced metabolic rates and obesity in humans [17, 18].

Obese individuals often display phenotypes consistent with the spectrum of non-alcoholic fatty liver disease (NAFLD), a condition characterized by abnormally high lipid content in liver tissues not due to the consumption of excess alcohol [19]. NAFLD is present in 20–30% of adults in developed countries, making it the most common liver disorder in this part of the world [20]. Prolonged accumulation of lipids in the liver can progress to inflammation and scarring, a stage of NAFLD known as non-alcoholic steatohepatitis (NASH). NASH, in turn, can progress to more serious hepatic fibrosis, decompensated cirrhosis, hepatocellular carcinoma, or liver failure [21, 22]. A number of factors can influence the progression of NAFLD, including insulin resistance, lifestyle (including nutrition), genetics, epigenetics, and the endocrine system [22, 23].

The effects of the endocrine system on liver health and obesity are of particular concern. Estrogen, for example, has a protective effect against NAFLD [23, 24], and women treated with the antiestrogen tamoxifen show a greater risk of developing NASH [25]. Estrogen also suppresses hepatic fibrosis by lessening activation of hepatic stellate cells in murine models [26]. Accordingly, estrogen replacement may reverse the progression of the disease in mice and humans who have mutations in the aromatase gene, which is involved in the synthesis of estrogens [27, 28]. Endocrine factors leading to NAFLD can also be epigenetic in nature. For example, prenatal exposure to tributyltin, a compound which inhibits aromatase and therefore estrogen synthesis, induces a phenotype similar to NAFLD in the offspring of exposed female mice as well as in the next two generations [29]. Dysregulation of the HPT axis, particularly a reduction in thyroid function and low levels of thyroid hormone, are also linked with NAFLD and NASH in animals and humans [23, 30].

Perchlorate (ClO_4_^-^) is a water-soluble anion and strong oxidizing agent with a variety of military and industrial applications, including rocket fuel, matches, fireworks, and airbags [31–33]. Due to its widespread use, high water solubility, and chemical stability, perchlorate has become widely distributed in the United States with contamination detected in at least 45 states [34], as well as in many other countries around the world. The compound has been documented in drinking water and in 213 of 285 common foods and drinks [35]. The U.S. Centers for Disease Control and Prevention also discovered perchlorate in the urine of all 2820 Americans tested with the highest levels found in children [36].

Due to ionic similarity of perchlorate to iodide, the perchlorate anion competitively inhibits the uptake of iodide at the sodium/iodide symporter (NIS) in thyroid follicular cells which can lead to decreased T_3_ and T_4_ levels and hypothyroidism [37]. Perchlorate exposure during development also has organizational effects on the vertebrate thyroid by leading to cellular hypertrophy, depleted colloid, and increased angiogenesis [38–40].

Our research team has employed the threespine stickleback (*Gasterosteus aculeatus*) fish model in many studies of perchlorate toxicity. For example, perchlorate impairs swimming performance and reproductive behaviors [41]. Perchlorate also affects normal androgen levels in embryos and disrupts gonadal development [40, 42, 43]. Because perchlorate disrupts the HPT axis, it is a prime candidate as a possible obesogen. Indeed, perchlorate increases lipid accumulation in developing stickleback; fish exposed to perchlorate during development form lipid droplets around thyroid tissue and these effects are not rescued with exogenous iodine [44]. Perchlorate exposure can also induce hepatic steatosis and cellular damage consistent with early onset of NAFLD in stickleback [45].

In this study, we investigate whether perchlorate exposure has obesogenic effects on developing zebrafish (*Danio rerio*) in order to determine if effects seen in stickleback are also found in another fish model. We exposed zebrafish to the same perchlorate concentrations as in our prior stickleback studies (plus an additional low concentration treatment) and reared these fish to reproductive maturity. We employed the same endpoints as used in stickleback to investigate NAFLD [45] as well as histology of whole-body sections and lipid specific staining to determine if lipids accumulate throughout the body.

## Materials and methods

All research protocols were approved by Northern Arizona University’s IACUC, protocol # 17–004. Fish were euthanized for histological preparation via overdose of buffered MS-222.

AB strain zebrafish embryos were collected directly after fertilization in tanks housing males and females at Northern Arizona University, mixed to disperse genetic diversity, and immediately distributed among 25 250ml glass jars. Each jar contained one of the four nominal perchlorate treatments (10μg/L, 10mg/L, 30mg/L, and 100mg/L) or control water (no perchlorate added, 0mg/L). Each concentration was replicated five times resulting in five jars containing 100ml each of four perchlorate treatments or the control. Each treatment solution also contained 500mg/L Instant Ocean for appropriate salinity and was prepared in reverse osmosis purified water. A perchlorate stock of 10g/L was created by dissolving 10g of sodium perchlorate in 1L of reverse osmosis purified water. This stock was diluted, based on the volume of water, to create the 10mg/L, 30mg/L, and 100mg/L perchlorate treatments. The 10μg/L treatment was created by dilution from the 100mg/L treatment.

Water was changed and dead embryos and larvae were removed daily for the first 25 days post-fertilization (dpf). After 25dpf, the fish were transferred to static 37.9L aquaria, filled to 2L of water per fish to keep fish density consistent across treatments. Each aquarium was equipped with an air stone and a sponge filter without the use of activated carbon. Each aquarium corresponded with an earlier 250ml jar, and fish count varied among tanks due to differences in mortality. Tanks of different treatments were arranged randomly on shelves in the experimental space to avoid positional associations between treatment and environmental conditions, and the ambient temperature was kept between 29.5°C and 32.5°C.

Larvae were fed live rotifers 3–4 times daily until large enough to consume pellet food. The pellet food (GEMMA Micro 300, Skretting) was introduced before this point, to prepare the larvae for the diet change. Once fully transitioned to pellet food (20dpf), fish were fed once a day. Water changes on static tanks occurred every two weeks because this interval maintains water pH and ammonia levels (<2mg/L) but also maintains prepared perchlorate concentrations [43]. Aquarium test strips were used to monitor pH and ammonia levels weekly, but the water chemistry never fell outside of “safe” parameters. At 133dpf, adult fish were euthanized with pH-neutral tricaine methanesulfonate (MS-222), weighed, and photographed laterally to obtain the standard length. Fish were transferred to 10% neutral buffered formalin for histological analysis.

Fixation of lipids was achieved using a procedure adapted from Tracy and Walia [46]. Incisions were made on the ventral sides of each fish to promote penetration of fixatives prior to treatment with a linoleic acid, lecithin, and ethylene glycol solution for five days. Samples were then rinsed several times with 70% ethanol over an eight-hour period and then rinsed in several changes of distilled water over an eight-hour period before transfer to a 2% aqueous chromic acid solution for 10 hours at 4°C. Specimens were then rinsed in several changes of distilled water and placed in 5% sodium bicarbonate for 24 hours at room temperature [46]. Lastly, specimens were rinsed in several changes of distilled water then dehydrated in a graded series of ethanol and xylene using a Shandon Citadel 2000 tissue processor (Leica Microsystems).

Fish were embedded individually in paraffin and sectioned at 10μm. Two ventral, two medial, and two dorsal histological sections were taken from each fish along the frontal plane. One slide from each anatomical position was stained with a standard Harris’ hematoxylin and eosin (H&E) protocol [47]. The other matching sections were prepared and stained with a modified protocol from Tracy and Walia [46]. Because our sections were larger than in Tracy and Walia, they were placed in Oil Red O for two hours on an agitating plate, then 85% propylene glycol for three minutes on an agitating plate, rinsed in water, and counterstained with Harris’ hematoxylin for one minute. Images of each slide were obtained using a Leica DM6 B digital microscope and Leica Application Suite X (LASX) software (Leica Microsystems). This software was also used to quantify the area occupied by lipids on each slide.

The sections stained with H&E were used for the analysis of liver lipid content to provide comparison with stickleback from Minicozzi et. al. (2019). Lipids in the liver were analyzed from the most anterior lobe, and areas with large arteries and veins were avoided. Because glycogen storage appears similar in color to lipid droplets when using this staining technique, a number of filters were applied in the Leica software to automate the analysis while ensuring that only lipid was counted. Because lipid droplets are generally round, all features with a roundness factor below 0.25 (where a value of 1 is a perfect circle) were excluded. All features smaller than 25 pixels (~5μm) were also filtered out to reduce noise. In some cases, manual adjustments were made to remove non-lipid structures not recognized by the filters. Representative images from the liver of each fish were evaluated according to three metrics by the Leica software: number of lipid droplets, median size of lipid droplets present in a section, and area occupied in the section by lipid droplets (total lipid area). All images were taken at 400x and the sample of liver completely filled the field of view resulting in an area of identical size captured for each individual. Because laboratory strains of zebrafish do not have genetic sex determination [48], H&E slides were used to determine the sex of each individual through identification of ovaries (female) or testes (male, S1 Fig).

In Oil Red O-stained slides, lipids in adipose tissue appeared bright red. To quantify the ratio of lipid content in the body (total body lipid content), the LASX software was set to pick bright red areas in sections from the ventral, medial, and dorsal sections of the entire fish. Because lipid deposits outside of the liver throughout the body and can occur in a variety of shapes and sizes, the only necessary filter used in this analysis excluded areas smaller than 100 pixels (~20μm). In some cases, the color threshold was manually adjusted to correct for slight variations in stain color. Images for analysis were taken at a magnification of 5x. The outline of each fish section was manually traced to give to total area of the fish’s body for each of the section types. To obtain the lipid content ratio of each section of the body, the total lipid area of each section was divided by the total area of the body. To calculate the total body lipid ratio, we added the lipid area of all three sections and the body area of all three sections (ventral, middle, and dorsal) and divided total body lipid area by the total body area.

All statistical tests were conducted with IBM SPSS statistics 24 (IBM Corp. 2016). Analysis of variance (ANOVA) was used to assess relationships between perchlorate exposure and mass, standard length, liver lipid droplet size, number of droplets, and total lipid droplet area. Differences across treatments in total body lipid ratios, as well as lipid ratios for the ventral, medial, and dorsal sections, were also examined using ANOVA. Sex ratio data were analyzed using a binomial test comparing the proportion of males in each treatment to 0.5 [48] to determine if perchlorate altered the sex ratio in developing zebrafish. All statistics were considered significant if p<0.05.

## Results

There was no difference in the mass of the fish across treatments (ANOVA, F_4,153_ = 1.06, p = 0.38, Table 1) but an ANOVA revealed a difference in the standard length (ANOVA, F_4,153_ = 2.64, p = 0.036, Table 1). When a Tukey’s post hoc was used, none of the groups showed a difference from each other (p>0.05 for all post hoc tests). The binomial tests revealed that the proportions of males in the 30mg/L (0.74) and 100mg/L (0.81) treatments were higher than the expected 50% sex ratio (p = 0.035 and 0.021 respectively, Table 1).

**Table 1:** Mean standard length and mass (+/- standard error of the mean) and sex ratio for the control treatment (0mg/L) and four perchlorate treatments.

Concentration	Standard Length (mm)	Mass (mg)	Sex Ratio (Male)	p-value
0mg/L	19.5 +/- 0.48	144 +/- 9.77	0.375	0.454
10μg/L	19.71 +/- 0.33	140 +/- 7.81	0.714	0.078
10mg/L	20.57 +/- 0.28	162 +/- 8.07	0.6	0.424
30mg/L	20.39 +/- 0.33	153 +/- 8.54	0.739	0.035*
100mg/L	19.05 +/- 0.57	140 +/- 12.54	0.813	0.021*

The p-value derives from a binomial test comparing the frequency of males to 0.5. Asterisk denotes a significantly higher proportion of males than 50%.

**Fig 1:**
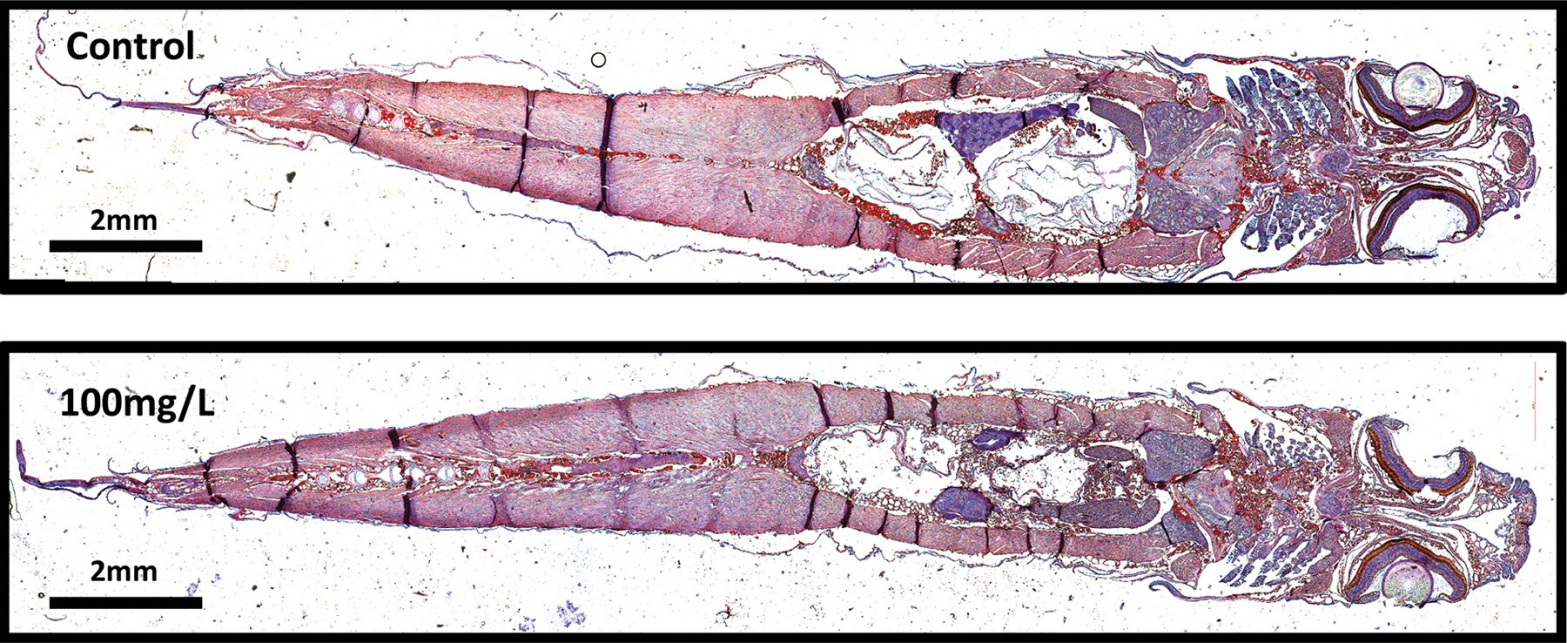
Whole-body lipid accumulation is not affected by perchlorate exposure in zebrafish. Lipid (bright red stain) appears around the gills, peritoneal cavity and around the spinal cord in both control and perchlorate treated zebrafish. Slides were frontally sectioned at 10μm and stained with oil red O and hematoxylin.

**Fig 2:**
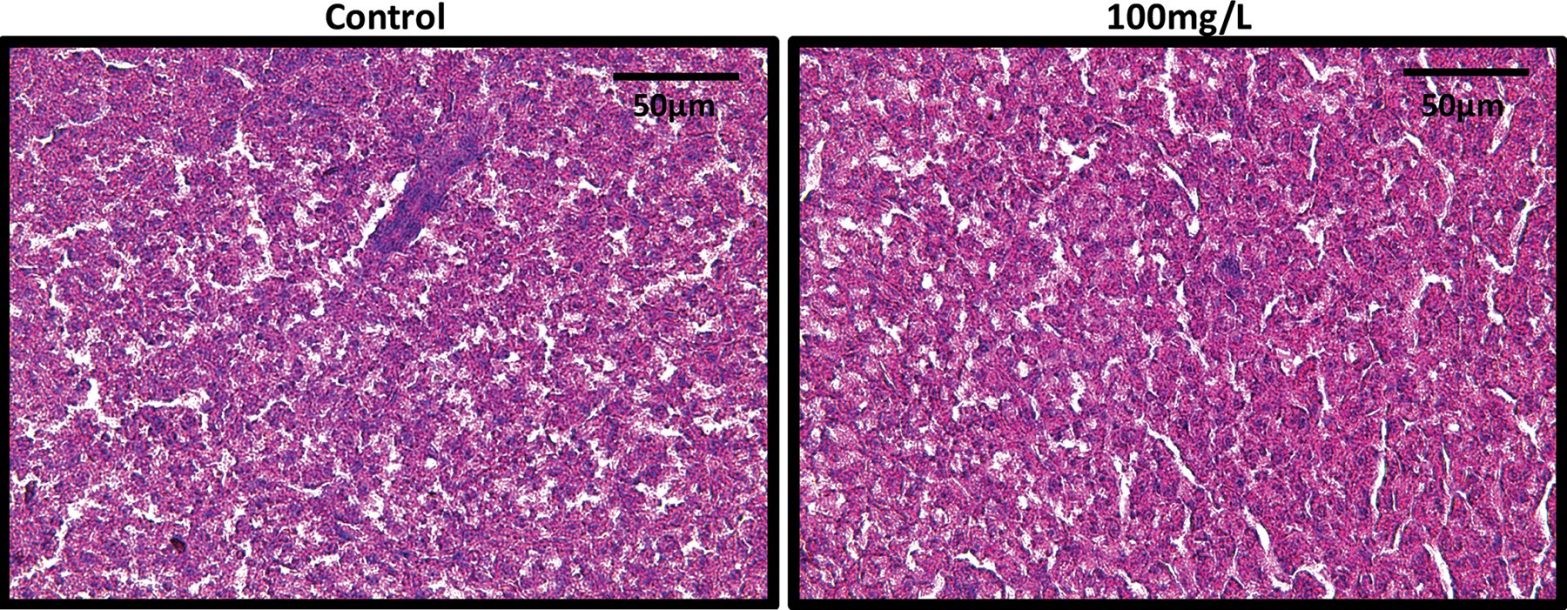
Lipids do not accumulate in zebrafish liver as a result of perchlorate exposure. Lipid droplets (white circles) appear throughout the liver, but neither control zebrafish nor perchlorate-treated zebrafish exhibit elevated lipid accumulation. Slides were stained with hematoxylin and eosin.

**Fig 3:**
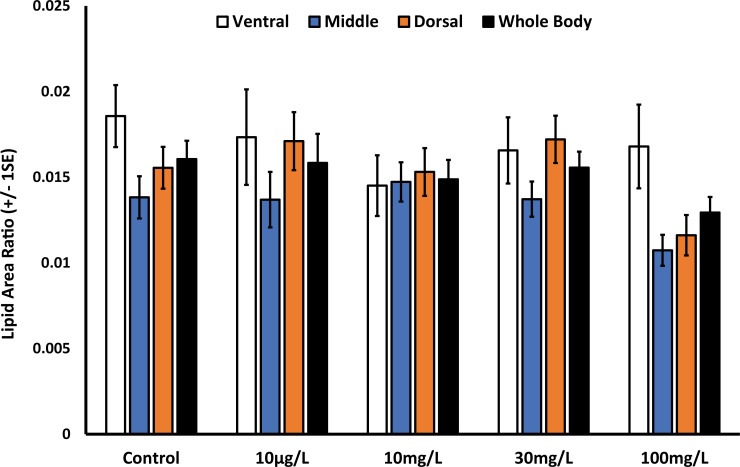
Control zebrafish and perchlorate exposed zebrafish do not differ in lipid content in the ventral, medial or dorsal regions of the body. Whole body lipid ratio is an average across all the sections of the fish.

Histologically, perchlorate appears to have no effect on either whole body lipid accumulation (Fig 1) or lipid accumulation in the liver (Fig 2). Control histological images appear strikingly similar to all of the perchlorate treated sections. Our data indicate no difference in the total body lipid content between perchlorate exposed fish and control fish (Fig 3, ANOVA, F_4,103_ = 1.0, p = 0.41). Similarly, there was no difference in body lipid content when comparing the ventral (ANOVA, F_4,108_ = 0.48, p = 0.75), medial (ANOVA, F_4,106_ = 1.49, p = 0.21), and dorsal (ANOVA, F_4,105_ = 1.26, p = 0.29) sections of perchlorate exposed fish to the control sections (Fig 3).

**Fig 4:**
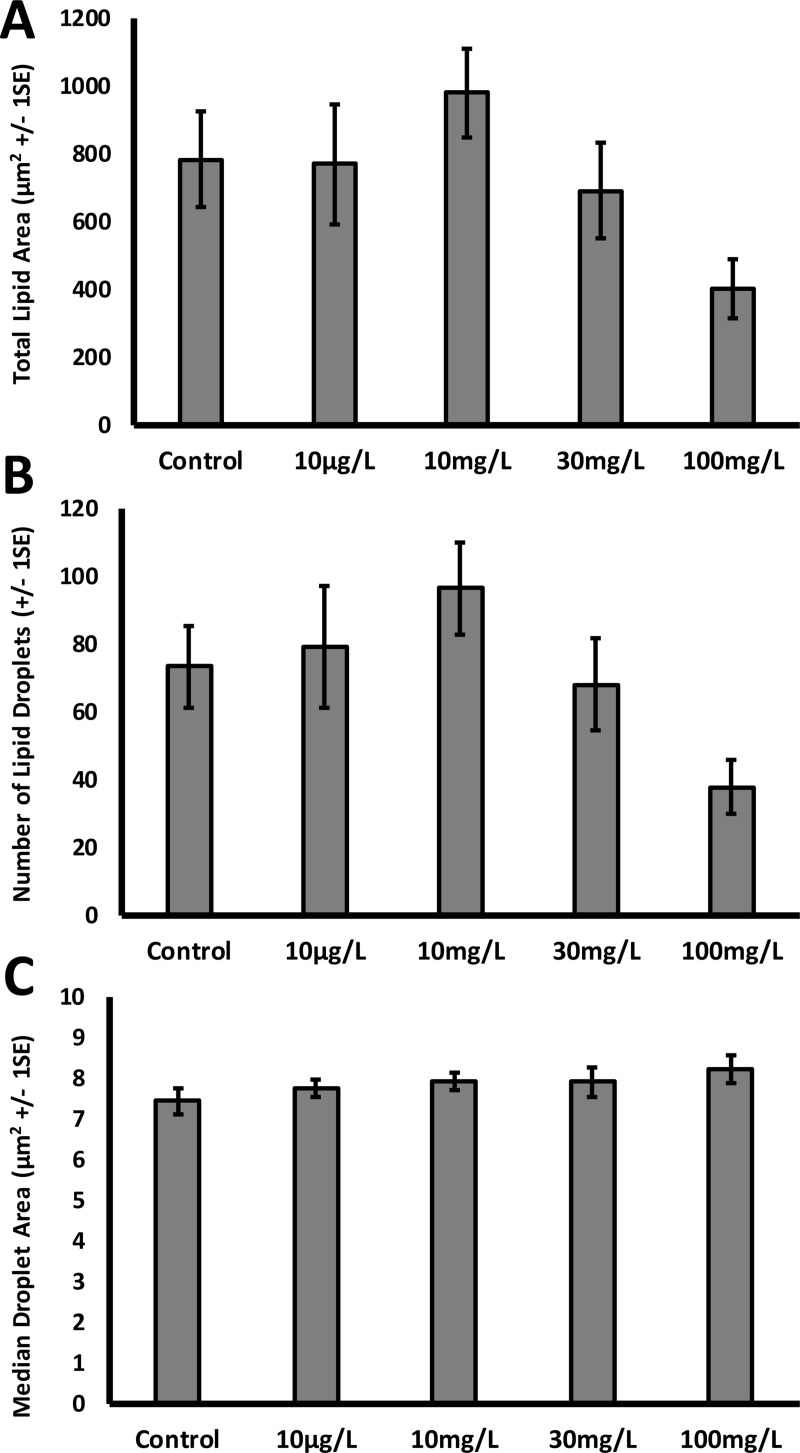
The livers of control and perchlorate-treated zebrafish do not differ in lipid accumulation, number of lipid droplets, or size of lipid droplets. A) Total lipid area in a section of liver. B) Number of lipid droplets in a section of the liver. C) Median size of lipid droplets in a section of the liver.

We also found no noticeable effect of perchlorate on liver morphology. Total lipid area did not differ between perchlorate-treated fish and controls (ANOVA, F_4,106_ = 1.69, p = 0.16), nor did the number of lipid droplets (ANOVA, F_4,106_ = 1.93, p = 0.11) or the median lipid area (ANOVA, F_4,106_ = 1.69, p = 0.16) (Fig 4).

## Discussion

**Fig 5:**
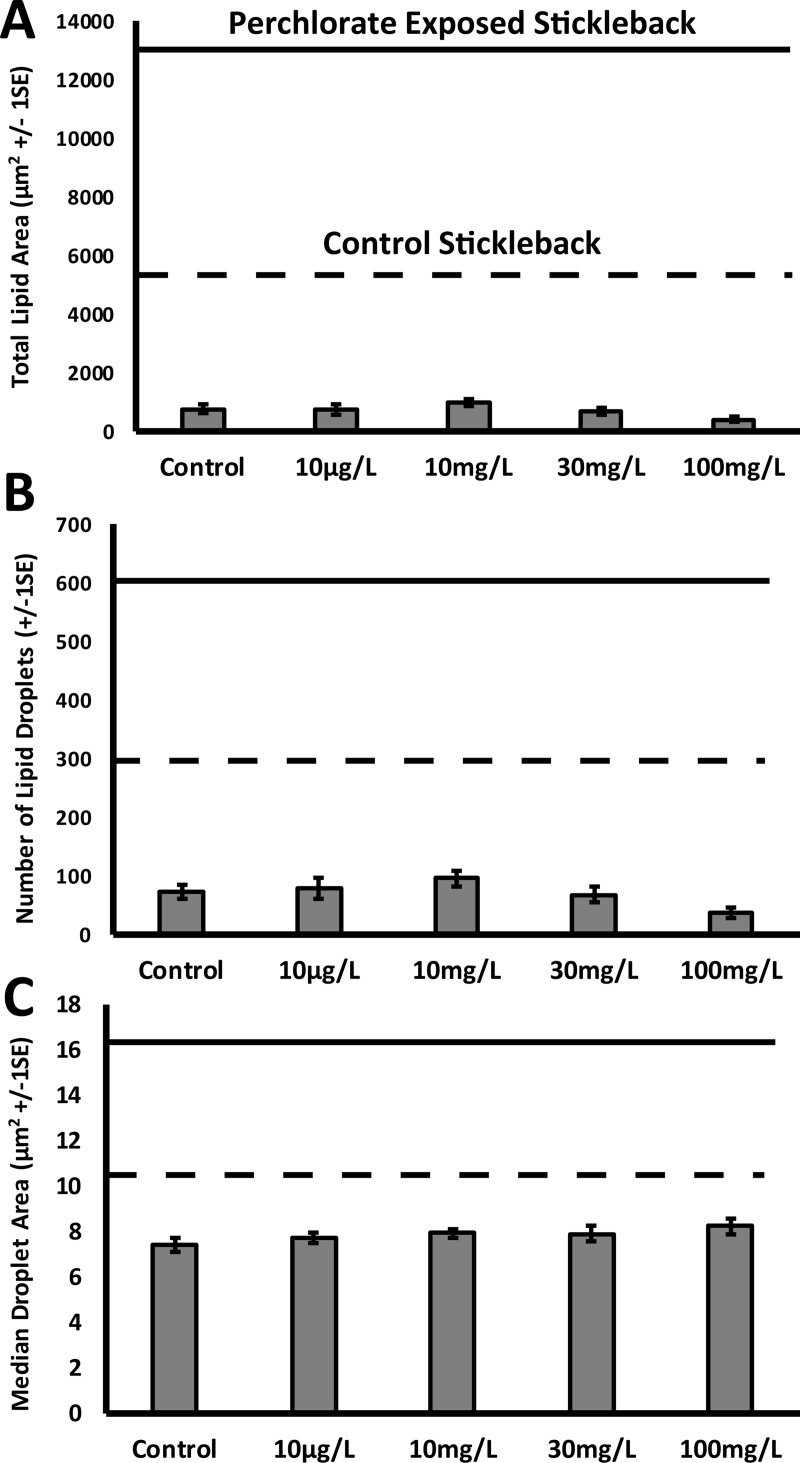
**Comparison of the control liver (dashed line) and perchlorate exposed liver (solid line) of threespine stickleback to zebrafish liver morphology (grey bars).** Stickleback data are derived from Minicozzi et. al. [45]. Perchlorate exposed line is an average value derived from treatment conditions in which stickleback were affected.

The liver of zebrafish did not show abnormal lipid accumulation at any perchlorate concentration tested, and fish in the control treatment did not differ from perchlorate-treated fish in mean lipid droplet size, number of lipid droplets present, or total lipid area. This contrasts with our findings in the threespine stickleback fish model (Minicozzi et al. 2019). Not only do the two species differ in their response to perchlorate exposure, but they also show differences in lipid content of the liver in control fish. The liver of stickleback in control conditions appears to have about six times more lipid content than does the liver in control zebrafish (Fig 5A). Similarly, stickleback livers have far more lipid droplets than do zebrafish livers (Fig 5B). In contrast, the size of lipid droplets in livers of both species appears to be similar (Fig 5C).

Given the dramatic effect of perchlorate exposure on the stickleback liver, we were surprised by the lack of response to perchlorate in the zebrafish liver. In some cases, stickleback liver lipid content doubled in response to perchlorate, whereas in zebrafish, we observed no noticeable change (Fig 5). Although liver physiology is thought to be conserved across vertebrates [49], the general structure of the liver differs among fishes. Stickleback tend to have a tubular arrangement of hepatocytes while cyprinids can vary between tubular or a solid arrangement of hepatocytes [50]. Some fishes (Gobeioidei and Tetraodontiformes) store lipids as droplets in the liver as a normal part of their physiology [50]. Other animal models show discrepancies in the onset and severity of NAFLD, which can be based on the species, sex, genetic strain, and/or diet [51–53]. Male C57/BL6 mice display less steatosis when compared to male Wistar rats when fed the same high-fat methionine choline-deficient diet (MCD) over the same time frame [52]. Male rats from three stains (Wistar, Long-Evans, and Sprague–Dawley) developed greater levels of steatosis when compared to the females of the same strains [52]. Male C57/BL6 mice can develop steatosis in four weeks [52], whereas other mouse strains (Diet-Induced Animal Model of NAFLD, DIAMOND) can develop the same pathologies in 8–16 weeks [54]. The mechanism of action by which perchlorate increases lipid content in stickleback liver is unknown and the mechanisms driving divergent responses in fishes warrant study.

Stickleback and zebrafish also diverge in the effects of perchlorate exposure on the thyroid. The stickleback response to perchlorate exposure during development includes hypertrophy of thyroid follicular cells and lipid accumulation and vascularization in the surrounding tissue [43]. Zebrafish also respond to perchlorate exposure through morphological changes to thyroid tissue but the obesogenic effect is not evident in the surrounding tissue [55]. Excess iodine rescues the developmental effects of perchlorate exposure on the stickleback thyroid, with the exception that lipid remains in surrounding tissue [43]. This suggests that lipid accumulation due to perchlorate exposure is not a direct effect of perchlorate on thyroid hormone levels. Interactions between sex hormone synthesis and the HPT axis [56] or epigenetic control of these systems may interact with perchlorate exposure to determine lipid accumulation in fishes.

Some EDCs influence gene expression, including expression of genes related to lipid storage [15, 16]. Tributyltin also has epigenetic effects [29], although it is unknown if these effects extend to genes related to lipid accumulation. Future investigations should test whether perchlorate causes obesogenic effects in some fishes, such as stickleback, by upregulating genes that promote fat storage.

Perchlorate treatment at the high exposure levels was associated with a sex ratio skewed toward males (Table 1). Sex determination in laboratory strains of zebrafish is environmentally mediated [57]. Other studies investigating the effects of perchlorate on sex ratios in zebrafish have shown a dose dependent trend towards feminizing gonadal sex [58] as opposed to our dose dependent shift towards masculinizing the gonadal sex. However, our results in this study are consistent with a masculinizing effect of perchlorate in stickleback [43, 59]. The effects of perchlorate on sexual development warrant further study, including the mechanisms driving divergent results.

The differing response to perchlorate exposure between stickleback and zebrafish may be due to differences in their evolutionary histories. Zebrafish are native to the Indian subcontinent [60], whereas stickleback are native to temperate, subarctic and arctic waters where lipid accumulation is a widespread adaptation for over-winter survival [61]. Testing the effects of perchlorate exposure on other fish species from both high and low latitude, including mechanistic studies at the molecular level, would address this hypothesis.

## Supporting information

S1 Fig Representative images of testes (A) and ovaries (B) used to determine the gonadal sex of each individual. Testes (A) stain light purple (spermatogonia) and dark purple (spermatocysts) and ovaries (B) stain light pink (large vitellogenic oocytes) and purple (previtellogenic oocytes). Gonadal sex was not determined from a small sample of the control (0mg/L, n = 3), 30mg/L (n = 4) and 100mg/L (n = 4) treatments because the gonads were not visible in the sections.
(TIF)

S1 Data (XLSX)
