# Supplementary figures and images for: Perchlorate exposure does not induce obesity or non-alcoholic fatty liver disease in zebrafish
Source: PLoS One. 2021 Aug 4;16(8):e0254500. doi: 10.1371/journal.pone.0254500 (PMC8336815; doi:10.1371/journal.pone.0254500)

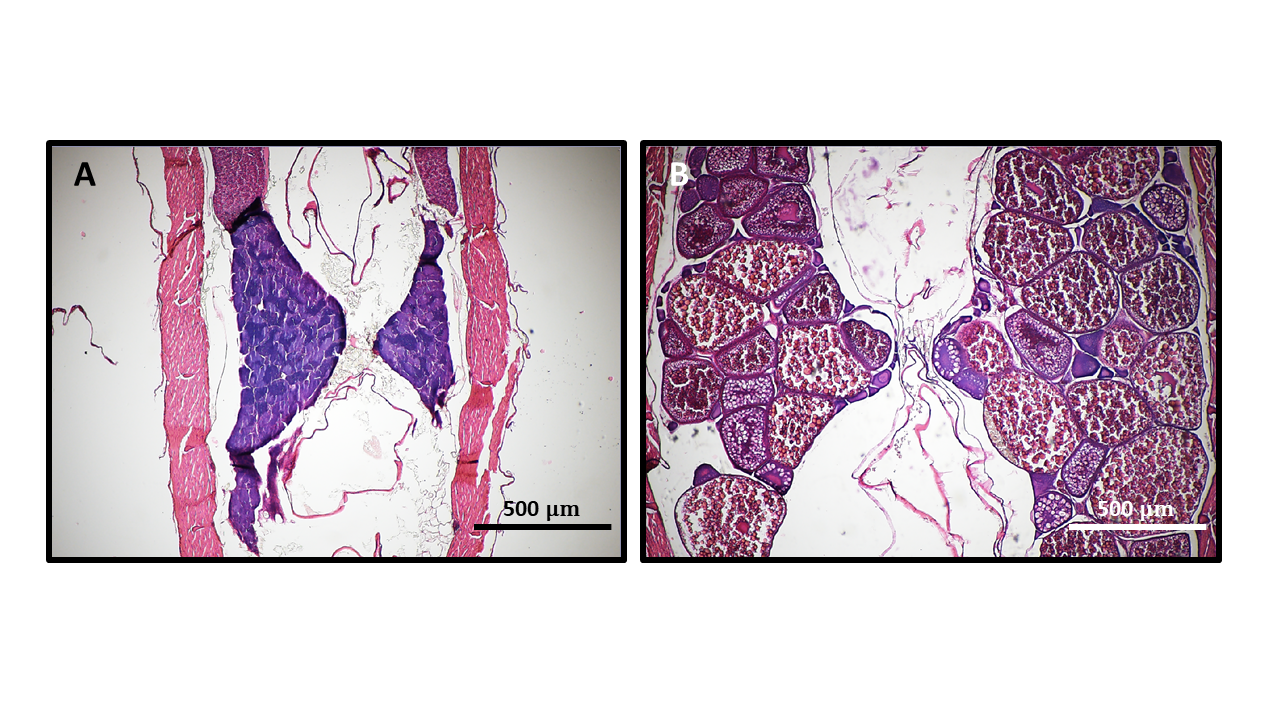

Supplement: S1 Fig — Representative images of testes (A) and ovaries (B) used to determine the gonadal sex of each individual. Testes (A) stain light purple (spermatogonia) and dark purple (spermatocysts) and ovaries (B) stain light pink (large vitellogenic oocytes) and purple (previtellogenic oocytes). Gonadal sex was not determined from a small sample of the control (0mg/L, n = 3), 30mg/L (n = 4) and 100mg/L (n = 4) treatments because the gonads were not visible in the sections. (TIF) [file pone.0254500.s001.tif]
